# Alcohol Screening on Admission to an Acute Mental Health Ward

**DOI:** 10.1192/bjo.2022.295

**Published:** 2022-06-20

**Authors:** Sutapa Gesell, Yvonne Tsitsiou, Josef Prochazka

**Affiliations:** Central and North West London NHS Foundation Trust, London, United Kingdom

## Abstract

**Aims:**

Alcohol misuse is estimated to cost the NHS £3.5 billion/year. Only 6% of people suffering from alcohol dependence in England, receive treatment per year, highlighting that alcohol misuse is under-identified. During the COVID-19 pandemic, people have significantly changed their drinking habits, evidenced by government tax receipt data, suggesting alcohol sales increased by 3% to 5% in the UK compared to 2019. Problems associated with harmful alcohol consumption were intensified by the crisis, even though the long-term impacts of COVID-19 on alcohol consumption are uncertain. There was a notable increase of patients with dual diagnosis of mental illness and alcohol misuse on our ward, which is a general adult inpatient psychiatric ward. As such, the aim was to assess and improve alcohol screening on admission to an acute mental health ward.

**Methods:**

Through a System One review, we assessed whether alcohol consumption is documented on admission (within 72 hours) in units, and a validated screening tool is used (AUDIT-C), which was expected in all patients. Their notes were initially retrospectively analysed and subsequently reviewed approximately six weeks following the implementation of interventions.

Interventions included presenting the findings of the primary survey to our colleagues during a multidisciplinary team meeting on the ward and a trust-wide audit meeting attended by both junior and senior doctors. Additional interventions included posters outlining the importance of alcohol screening in the interview rooms of the acute wards (including a QR code link to our presentation and findings).

**Results:**

Out of the 17 patients on the ward, 47% (8/17) were not appropriately screened for alcohol misuse during their first 72 hours of admission. 47% (8/17) had no documented alcohol history on admission clerking. Only 12% (2/17) had partially quantifiable alcohol intake, both drinking above the recommended weekly amount. None of the ‘Current Drinker’ patients had AUDIT-C screening. Improvement was noted following the interventions during the secondary survey.

**Conclusion:**

Although alcohol screening in acute psychiatric admissions is often vague or incomplete, simple reminders and education can improve screening. If the alcohol history cannot be obtained from the patient on admission, which is often the case, the clinician should clearly document review of notes for historical alcohol use, to avoid potential complications, such as alcohol withdrawal, delirium tremens or seizures.

This project raises further questions on how effective brief interventions for excessive alcohol consumption in acutely unwell/psychotic patients are, encouraging a further area of research.

